# Outcomes of Novel Hormonal Therapies in Men With Advanced Prostate Cancer by Treating Specialist

**DOI:** 10.1002/cam4.71219

**Published:** 2025-09-09

**Authors:** Kassem S. Faraj, Mary Oerline, Samuel Kaufman, Avinash Maganty, Megan E. V. Caram, Vahakn B. Shahinian, Brent K. Hollenbeck

**Affiliations:** ^1^ Dow Division of Health Services Research University of Michigan Ann Arbor Michigan USA; ^2^ VA Health Services Research, Centers for Clinical Management Research, VA Ann Arbor Healthcare System Ann Arbor Michigan USA; ^3^ Division of Oncology, Department of Internal Medicine University of Michigan Ann Arbor Michigan USA; ^4^ Division of Nephrology, Department of Internal Medicine University of Michigan Ann Arbor Michigan USA; ^5^ Department of Urology Massachusetts General Hospital Boston Massachusetts USA

**Keywords:** adverse events, financial toxicity, prostate cancer, quality

## Abstract

**Introduction:**

In the past decade, the management of advanced prostate cancer has shifted to novel hormonal therapies. As a result, urologists have increased their involvement in the management of advanced prostate cancer. These therapies require close monitoring due to the possibility of adverse cardiometabolic events. We assessed outcomes among men diagnosed with advanced prostate cancer started on novel hormonal therapy by a urologist compared to those by a medical oncologist.

**Methods:**

We performed a retrospective cohort study of Medicare beneficiaries with advanced prostate cancer treated with a novel hormonal therapy between 2012 and 2019. The primary outcome was an adverse event comprised of a hospital visit for a cardiometabolic event within 6 months of starting a novel hormonal therapy. Secondary outcomes included monthly out‐of‐pocket costs and treatment adherence.

**Results:**

There were 1212 (23%) and 4124 (77%) patients who were prescribed a novel hormonal therapy for the first time by a urologist and medical oncologist, respectively. No difference in the composite adverse event measure was observed in those managed by urologists or medical oncologists (4.2% vs. 4.7%, respectively, *p* = 0.49). Out‐of‐pocket costs, in men without low‐income subsidies, did not vary by specialty ($772 vs. $790, *p* = 0.58). Adherence to treatment did not vary in men managed by urologists or medical oncologists (75% vs. 74%, respectively, *p* = 0.64).

**Conclusions:**

The specialty of the physician prescribing a novel hormonal therapy was not associated with the risk of a cardiometabolic adverse event. Further, management by a urologist did not adversely affect costs to patients or adherence.

## 1 Introduction

Advanced prostate cancer accounts for approximately 35,000 deaths annually in the United States [1]. Beyond chronic androgen deprivation, the treatment paradigm has shifted over the past decade. Once limited to physician‐administered chemotherapy (e.g., docetaxel), the field now predominantly involves oral medications, such as those that inhibit androgen biosynthesis or androgen receptors. Novel hormonal therapies have demonstrated efficacy similar to chemotherapy and are associated with fewer serious and potentially life‐threatening toxicities [2, 3, 4, 5]. Nonetheless, by manipulating the androgen axis, patients can experience significant metabolic and cardiovascular adverse events [6]. Further, oral therapies are significantly more expensive and are associated with high costs to patients [7, 8], which can be associated with treatment nonadherence [9, 10].

The convenience of oral administration and relative tolerability of oral therapies have resulted in a growing role for urologists in managing advanced prostate cancer [11], a disease traditionally under the purview of medical oncologists. This evolving care model is potentially a double‐edged sword. On one hand, urologists' focus on prostate cancer and their longstanding relationship with the patient, often since diagnosis, may reduce care fragmentation and improve treatment adherence. On the other hand, urologists lack formal training in managing medical complications of these therapies. Indeed, experience with a wide variety of advanced malignancies and focused recommendations from professional guidelines [12] provide medical oncologists with skills that potentially make them better suited to handle the broader psychosocial and medical needs of these men.

For these reasons, we assessed outcomes among men diagnosed with advanced prostate cancer who were started on novel hormonal therapy by a urologist compared to those by a medical oncologist. Due to differences in medical training between the specialties, we hypothesized that men managed by urologists would have more adverse events. Further, because of their experience in navigating financial issues for a much larger group of patients with diverse diagnoses, we hypothesized that men managed by medical oncologists would have lower drug‐related costs, which would also be associated with higher rates of treatment adherence.

## 2 Methods

This was a retrospective cohort study that used a 20% sample of men with Traditional Medicare from 2012 through 2019 diagnosed with advanced prostate cancer. Men with advanced prostate cancer were identified using information on their use of androgen deprivation therapy, as previously described [6, 13]. Briefly, we categorized men to be on androgen deprivation chronically as those who had a history of bilateral orchiectomy or had continuously been on > 6 months of androgen deprivation therapy [6]. Patients receiving androgen deprivation therapy for local therapy were excluded. The study population was limited to men who were at least 66 years of age, allowing for a 12‐month look back to assess comorbidities in the pre‐treatment period, to ensure the absence of associated local treatment and to exclude men receiving treatment with one of the novel hormonal therapies prior to starting conventional androgen deprivation. We also limited our cohort to men receiving a novel hormonal therapy (i.e., abiraterone or an androgen receptor inhibitor [enzalutamide, apalutamide, darolutamide]) for the first time between January 1, 2012 and December 31, 2019, using Medicare Part D claims. The primary exposure was the specialty of the prescribing physician (urology versus medical oncology) associated with the first claim for a novel hormonal therapy.

### 2.1 Outcomes

Our primary outcome was a measure consisting of an admission to a hospital or a visit to the emergency department for a cardiovascular or metabolic event, assessed for the 6‐month period after starting a novel hormonal therapy [6]. Only those with a primary diagnosis code commonly associated with known adverse events for these therapies were included. Secondarily, adverse events were assessed separately by group (i.e., diabetes, hypertension, cardiac). To establish temporal precedence and more tightly link therapy to the adverse event, only those events in patients without a preexisting diagnosis in the year before initiating the therapy were included.

Secondary outcomes included monthly patient out‐of‐pocket costs and treatment adherence. A patient's out‐of‐pocket costs were determined by summing the “patient pay amount” the first 6 months after treatment initiation divided by the days on treatment. This was then multiplied by 30 [7]. The resulting figure was the out‐of‐pocket quantity a patient paid, on average, each month. Out‐of‐pocket costs were assessed excluding those with low‐income subsidies (*n* = 1260 (24%)), as out‐of‐pocket costs in this context are often minimal and would significantly skew the results. Treatment adherence was determined based on the percentage of days a patient had available prescription fills over a period of 6 months (i.e., the “proportion of days covered”) [7, 14]. This was calculated by calculating the number of days a prescription was supplied for a 6‐month period divided by 180 days. Per convention, adherence was defined as a percentage of days covered by a prescription of 80% or greater in the first 6 months of initiating treatment. Because treatment adherence and out‐of‐pocket costs were determined based on a 6‐month follow‐up period, we excluded men who died within 6 months of starting therapy from these analyses.

### 2.2 Statistical Analysis

We compared the characteristics of patients managed by urologists and medical oncologists with a Chi‐squared test. Next, we fit multivariable logistic regression models to evaluate the association between physician specialty and the presence of a composite adverse event within 6 months of starting a novel hormonal therapy for the first time. These models were adjusted for age, race, rural residence, socioeconomic class [15], comorbidities [16], time on androgen deprivation therapy, calendar year, and market variables. A similar modeling framework was used to assess treatment adherence. For monthly out‐of‐pocket costs, we fit a generalized linear model to assess the effect of specialty on this outcome. Subgroup analyses were performed to assess for heterogeneity by drug type (i.e., androgen biosynthesis inhibitors or androgen receptor inhibitors) for adverse events, costs, and treatment adherence. All models were adjusted for the same covariates above. Sensitivity analyses were performed assessing all outcomes in the year after therapy initiation.

Analyses were conducted using STATA 17 (College Station, TX). We calculated predicted outcomes using the margins command in STATA. All tests were two‐sided, with the probability of a type 1 error established at 0.05. The institutional review board at our institution deemed this study exempt from review.

## 3 Results

**FIGURE 1:**
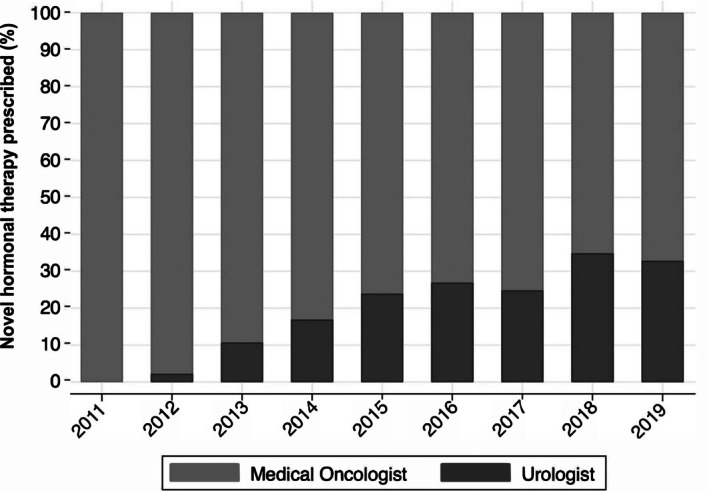
First time prescriptions for novel hormonal therapies for advanced prostate cancer by physician specialty. The prescriptions by urologists increased over time, ranging from 2% in 2012 to 33% in 2019 (*p* value for time trend < 0.001).

There were 5336 patients with advanced prostate cancer who received a novel hormonal therapy for the first time between 2012 and 2019. Of these, 1212 (23%) and 4124 (77%) were prescribed their first therapy by a urologist and medical oncologist, respectively. The prescriptions by urologists increased over time (Figure 1), ranging from 2% in 2012 to 33% in 2019 (*p* value for time trend < 0.001). Men managed by urologists were on androgen deprivation for a longer period before initiating treatment with a novel hormonal therapy (1135 days vs. 954 days, *p* < 0.001). Otherwise, differences in characteristics between specialties were modest (Table 1). However, those managed by urologists were more often started on an androgen receptor inhibitor compared to those managed by a medical oncologist (63% vs. 34%, *p* < 0.001). Primary and secondary outcomes did not differ by specialist on unadjusted analyses (Figures S1–, S3).

**TABLE 1:** Characteristics of patients managed by urologists and medical oncologists who receive oral targeted therapies for advanced prostate cancer.

	Urologist	Medical oncologist	*p*
Number of beneficiaries	1212	4124	
Mean age (SD)	81 (7)	80 (7)	< 0.001
Race			
White	989 (82)	3488 (85)	< 0.001
Black	170 (14)	409 (10)	
Other	48 (4)	210 (5)	
Comorbidity index			
0	546 (45)	1739 (42)	0.32
1	213 (18)	751 (18)	
2	193 (16)	722 (18)	
3+	260 (21)	903 (22)	
Hospital bed per 100,000			
Low	375 (31)	1261 (31)	0.15
Intermediate	404 (33)	1490 (36)	
High	428 (36)	1356 (33)	
Med adv penetration			
Low	428 (35)	1374 (34)	0.059
Intermediate	400 (33)	1287 (31)	
High	379 (31)	1439 (35)	
Socioeconomic status (%)			
Low	386 (32)	1235 (30)	0.10
Medium	417 (34)	1358 (33)	
High	409 (34)	1531 (37)	
Rural (%)	235 (19)	987 (24)	0.001
Time on ADT until drug start			
Mean (SD)	1135 (864)	954 (752)	< 0.001
Median (IQR)	989 (411, 1709)	812 (376, 1357)	< 0.001
Low‐income subsidy (%)	256 (21)	1004 (24)	0.020

**FIGURE 2:**
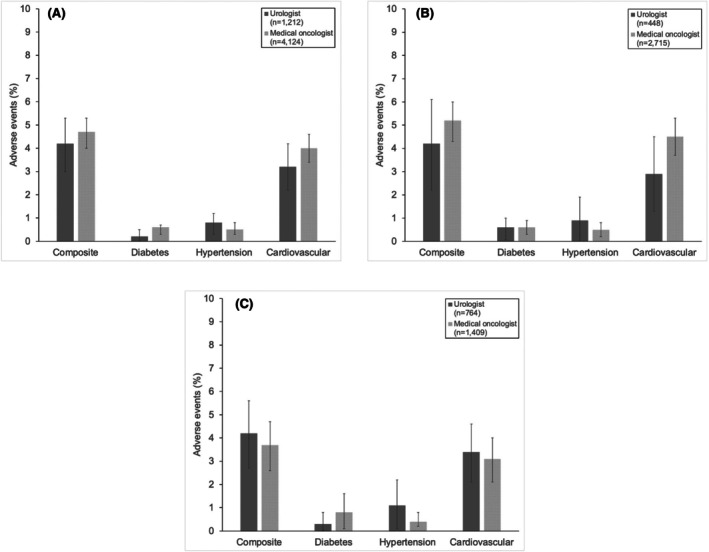
Adverse events according to prescribing physician. (A) Among all men started on a novel hormonal therapy, there was no difference in the percentage of men suffering an adverse cardiometabolic event between those managed by urologists and medical oncologists (4.2% vs. 4.7%, respectively, *p* = 0.49). (B) In men prescribed an androgen biosynthesis, the percentage of cardiometabolic adverse events did not differ between prescriptions originating from a urologist and medical oncologist (4.2% vs. 5.2%, respectively, *p* = 0.40). (C) In men prescribed an androgen receptor inhibitor, the percentage of cardiometabolic adverse events did not differ between prescriptions originating from a urologist and medical oncologist (4.2% vs. 3.7%, respectively, *p* = 0.30).

**FIGURE 3:**
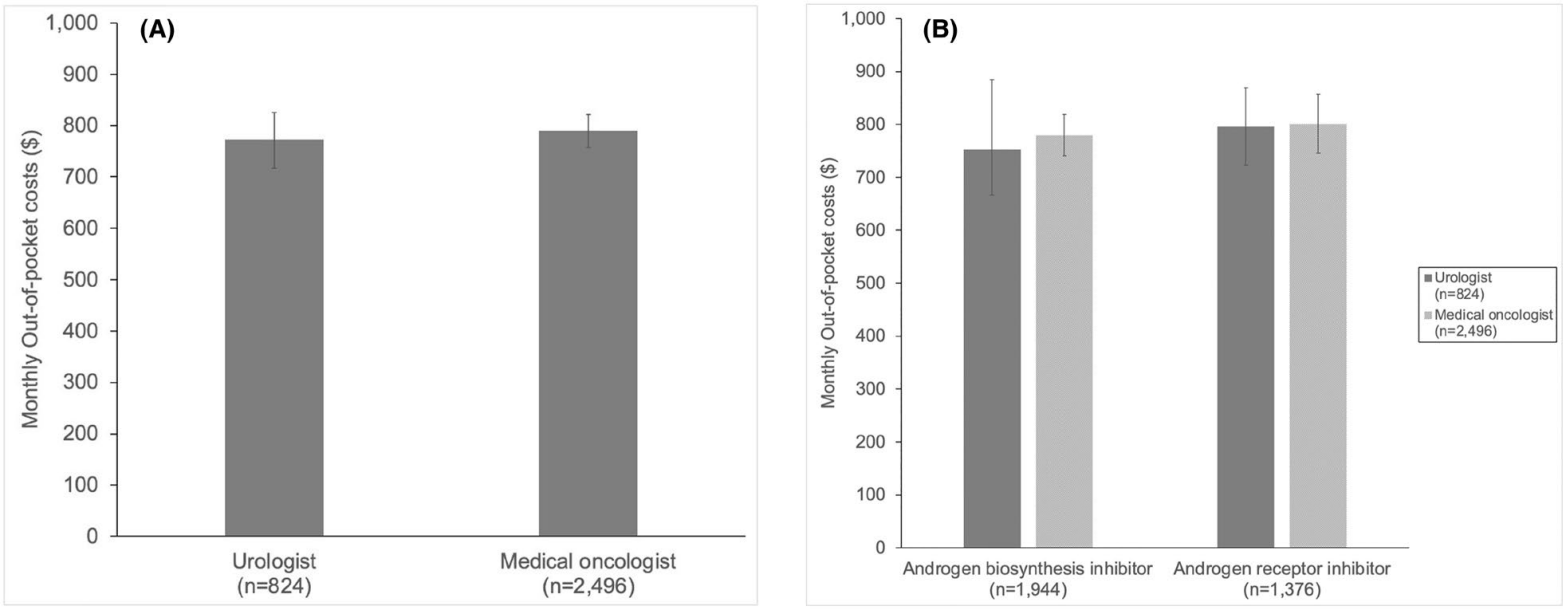
Out‐of‐pocket costs for men without low‐income subsidies. (A) Out‐of‐pocket costs did not vary between men managed by urologists and medical oncologists ($772 vs. $790, respectively, *p* = 0.58). (B) Out‐of‐pocket costs did not differ between prescriptions originating from a urologist or medical oncologist in those prescribed an androgen biosynthesis inhibitor ($753 vs. $780, respectively, *p* = 0.58) or an androgen receptor inhibitor ($796 vs. $701, respectively, *p* = 0.91).

**FIGURE 4:**
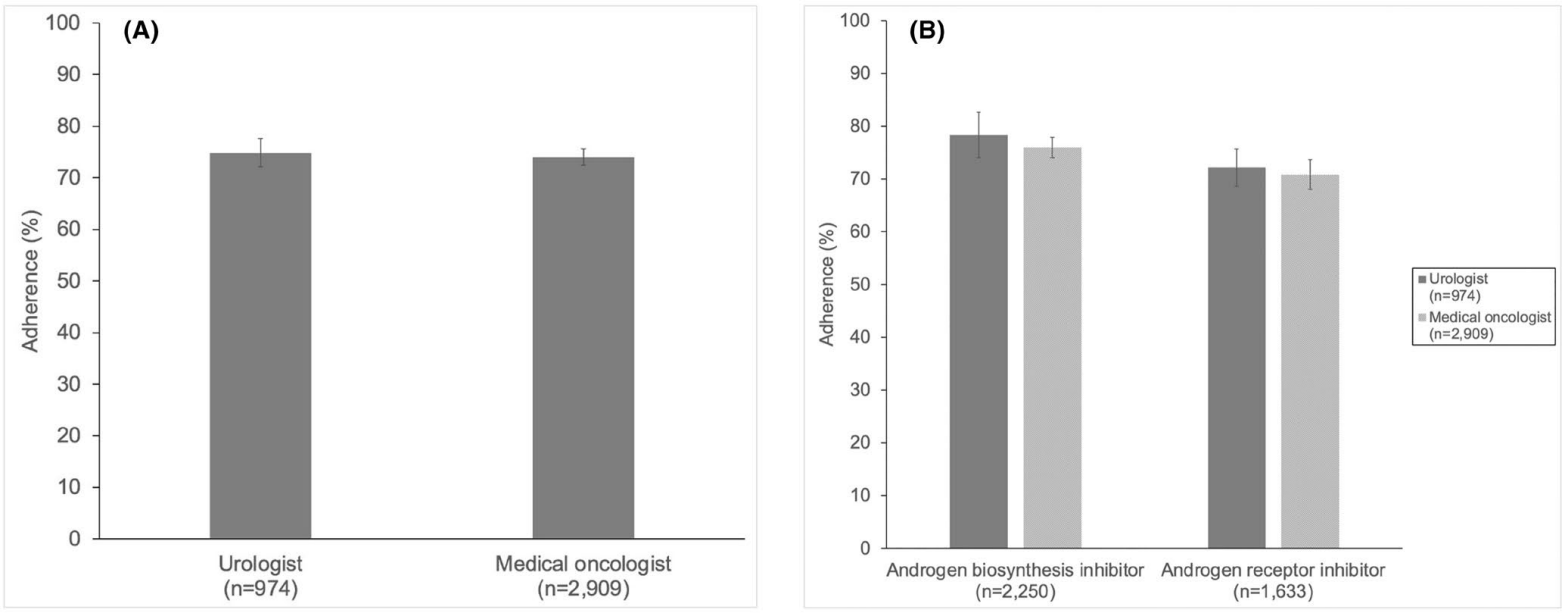
Adherence to treatment by prescribing physician specialty. (A) Treatment adherence did not vary in men managed by urologists and medical oncologists (75% vs. 74%, respectively, *p* = 0.64). (B) Adherence to treatment did not vary in men managed by urologists and medical oncologists for those prescribed an androgen biosynthesis inhibitor (78% vs. 76%, respectively, *p* = 0.34) or an androgen receptor inhibitor (72% vs. 71%, respectively, *p* = 0.57).

There were no differences in the composite measure for adverse events between men managed by urologists and medical oncologists (Figure 2A; 4.2% vs. 4.7%, *p* = 0.49). Out‐of‐pocket costs also did not vary between specialties (Figure 3A; $772 vs. $790, *p* = 0.58). Adherence to treatment also did not vary in patients who were managed by urologists and medical oncologists (Figure 4A; 75% vs. 74%, *p* = 0.64). Sensitivity analyses assessing adverse events, out‐of‐pocket costs, and treatment adherence within 12 months of initiating therapy demonstrated no differences in these outcomes by specialty (Figures S4–, S6).

In the subgroup analysis, composite adverse events did not vary by specialty in those who were started on an androgen biosynthesis inhibitor (Figure 2B; composite 4.2% vs. 5.2%, *p* = 0.40) or an androgen receptor inhibitor (Figure 2C; composite 4.2% vs. 3.7%, *p* = 0.30). Out‐of‐pocket costs (Figure 3B) for those without low‐income subsidies did not differ by specialty for those who were started on an androgen biosynthesis inhibitor ($753 vs. $780, *p* = 0.58) or an androgen receptor inhibitor ($796 vs. $701, *p* = 0.91). Treatment adherence (Figure 4B) also did not vary by prescribing specialist for those prescribed an androgen biosynthesis inhibitor (78% vs. 76%, *p* = 0.34) or an androgen receptor inhibitor (72% vs. 71%, *p* = 0.57).

## 4 Discussion

The specialty of the physician prescribing a novel hormonal therapy in men with advanced prostate cancer was not associated with a cardiometabolic adverse event. Further, the specialty of the prescribing physician was not associated with greater costs to patients, suggesting that urologists are facile at navigating the complex milieu of patient financial support programs. Patient adherence to planned treatment also did not differ between prescribing specialists. Collectively, these data support the role of urologists in managing advanced prostate cancer, at least by these measures.

As new novel hormonal therapies are more convenient for patients and have fewer severe side effects, urologists have played a growing role in men with advanced prostate cancer. Potential benefits of this evolution in cancer delivery include reducing delays in treatment initiation (i.e., due to referral), maintaining continuity of care, and avoiding the consequences of care fragmentation, such as duplicative testing [17, 18]. However, novel hormonal therapies are associated with significant cardiometabolic events (e.g., major adverse cardiac events, metabolic abnormalities) in clinical trials [2, 19] and real‐world data [6], supporting the need for regular monitoring. This is likely related to the effect of these medications on the androgen pathway, where prior work has demonstrated increased incidence of cardiovascular and metabolic events in men with advanced prostate cancer treated with androgen deprivation therapy [20, 21, 22]. The lack of internal medicine training for a urologist and the specialized nature of their practice raise concerns about patient quality and safety. For example, men on chronic androgen deprivation therapy managed by urologists are less likely to undergo bone mineral density testing relative to those with medical oncology or primary care involved in their care [23, 24]. In this study, the specialty of the prescribing physician was not associated with a cardiometabolic adverse event, suggesting that training differences may be inconsequential in this context.

Although novel hormonal therapies are convenient, their high costs are often passed down to the patient, which can exceed $10,000 annually [8]. Such costs may contribute to financial toxicity, which can manifest as depression, anxiety, and decreased spending on non‐healthcare‐related necessities [25, 26]. Some may cope with these financial consequences through treatment non adherence and abandonment [25]. Medical oncologists manage a broad variety of cancers that often involve expensive specialty drugs. As a result, their professional guidelines have recommended specific guidance on discussing costs of therapy and assessing the comparative value of treatment options with patients [12]. We found that urologists tended to prescribe androgen receptor inhibitors, which are newer and do not have less expensive alternatives (e.g., generic abiraterone). However, there was no difference in the out‐of‐pocket costs between patients managed by urologists and medical oncologists. Additionally, adherence to treatment—which could be influenced by multiple factors including costs of therapy, adverse events, access to therapy—did not vary between specialists. These findings suggest that patients who are started on therapies by urologists are not at increased risk for adverse financial outcomes or premature treatment discontinuation.

This study has a few limitations that should be considered in the context of interpreting the results. First, we were unable to account for disease severity, including the presence of or burden of metastatic disease. This is particularly relevant given that there may be an aspect of patient selection in play when considering the complexity of patients seen by medical oncologists vs. urologists. However, we included the duration on chronic androgen deprivation therapy in our models, which may be an indicator of disease severity. Further, until 2018, all novel hormonal therapies were approved for only the metastatic‐castrate resistant setting. Additionally, patient demographics and comorbidities did not significantly differ between groups, making it unlikely that meaningful differences in non‐cancer comorbidities existed between the groups. Second, we assessed outcomes in the first 6 months after the first novel hormonal therapy was filled. If patients were sequenced on a subsequent treatment and experienced an adverse event beyond this time period, we would not have captured this in our analysis. However, extending the follow‐up period to 12 months revealed no difference in adverse events. Therefore, we would not expect differences between specialties beyond this time period. Further, the median time on therapy in this cohort was 8 months. This suggests that we would expect to identify most adverse events in the intervals we assessed. Next, due to differences in care models between countries and incentive structures, these findings would likely not be generalizable to other countries. Finally, we assigned patients to a specialist based on the first prescription for novel hormonal therapy. It is possible that there was multidisciplinary care between medical oncologists and urologists in managing these patients that we did not account for. However, prior work has demonstrated that most prescriptions from urologists arise from those who practice out of single‐specialty groups, where the urologist would likely be the predominant cancer provider after the initial prescription is made [11].

This study provides reassurance regarding the growing role of the urologist in the management of advanced prostate cancer. Nonetheless, it is important that urologists recognize clinical scenarios in which medical oncology referral is warranted, including high‐volume disease suitable for triplet therapy and those with disease progression on therapy that requires escalation to cytotoxic therapy [27, 28]. Further, understanding the optimal novel hormonal therapy duration should be considered to avoid prolonging therapy to a point where a patient may become ineligible for chemotherapy. This is particularly pertinent to this older cohort, who often have multiple comorbidities that may be exacerbated by a longer duration on therapy that subsequently precludes the use of docetaxel or cabazitaxel. In those at higher risk for adverse cardiac events, early involvement of cardiologists in cardiac oncology clinics may be useful in monitoring and reducing the risk of these events.

## 5 Conclusions

In men using a novel hormonal therapy for advanced prostate cancer for the first time, physician specialty was not associated with a cardiometabolic adverse event, out‐of‐pocket costs, or treatment nonadherence. These data support the increasing role of urologists in managing advanced prostate cancer. However, future work should explore other dimensions of quality, such as appropriateness and disease response rates.

## Author Contributions

**Kassem S. Faraj:** conceptualization (equal), formal analysis (equal), investigation (equal), methodology (equal), supervision (equal), writing – original draft (equal), writing – review and editing (equal). **Mary Oerline:** data curation (equal), formal analysis (equal), software (equal), writing – review and editing (equal). **Samuel Kaufman:** data curation (equal), formal analysis (equal), resources (equal), software (equal), writing – review and editing (equal). **Avinash Maganty:** investigation (equal), validation (equal), writing – review and editing (equal). **Megan E. V. Caram:** conceptualization (equal), investigation (equal), supervision (equal), writing – review and editing (equal). **Vahakn B. Shahinian:** conceptualization (equal), funding acquisition (equal), methodology (equal), project administration (equal), supervision (equal), validation (equal), writing – review and editing (equal). **Brent K. Hollenbeck:** conceptualization (equal), funding acquisition (equal), investigation (equal), project administration (equal), resources (equal), supervision (equal), visualization (equal), writing – review and editing (equal).

## Conflicts of Interest

The authors declare no conflicts of interest.

## Supporting information


**Figure S1:** Unadjusted adverse events by specialist (*p < 0.05). (A) Entire cohort. (B) Androgen biosynthesis inhibitor. (C) Androgen receptor inhibitor.

**Figure S2:** Unadjusted treatment adherence by specialist (*p < 0.05). (A) Entire cohort. (B) By drug type.

**Figure S3:** Unadjusted patient out‐of‐pocket costs by specialist in men without low‐income subsidies (*p < 0.05). (A) Entire cohort. (B) By drug type.

**Figure S4:** Adverse events by specialty in the 12 months after treatment initiation. There was no difference in the composite adverse event (7% vs. 8%, *p* = 0.43) or any individual adverse events between specialties.

**Figure S5:** Monthly out‐of‐pocket costs in the 12 months after treatment initiation. There was no difference in out‐of‐pocket costs between specialties ($710 vs. $740, *p* = 0.30).

**Figure S6:** Adherence to treatment in the 12 months after treatment initiation. There was no difference in adherence between specialties (63% vs. 60%, *p* = 0.20).

## Data Availability

The data used in this study were Medicare claims data from the Centers for Medicare and Medicaid Services. The data is provided under license/permission by CMS and may be shared upon request, if permissible by CMS.
