# Supplementary material for: Outcomes of Novel Hormonal Therapies in Men With Advanced Prostate Cancer by Treating Specialist
Source: Cancer Med. 2025 Sep 9;14(17):e71219. doi: 10.1002/cam4.71219 (PMC12417965; doi:10.1002/cam4.71219)
Supplement: Supplementary file 1 — Figure S1: Unadjusted adverse events by specialist (*p < 0.05). (A) Entire cohort. (B) Androgen biosynthesis inhibitor. (C) Androgen receptor inhibitor. [file CAM4-14-e71219-s004.docx]

**Supplemental Figure 1**. Unadjusted adverse events by specialist (*p<0.05). A) Entire cohort. B) Androgen biosynthesis inhibitor. C) Androgen receptor inhibitor.


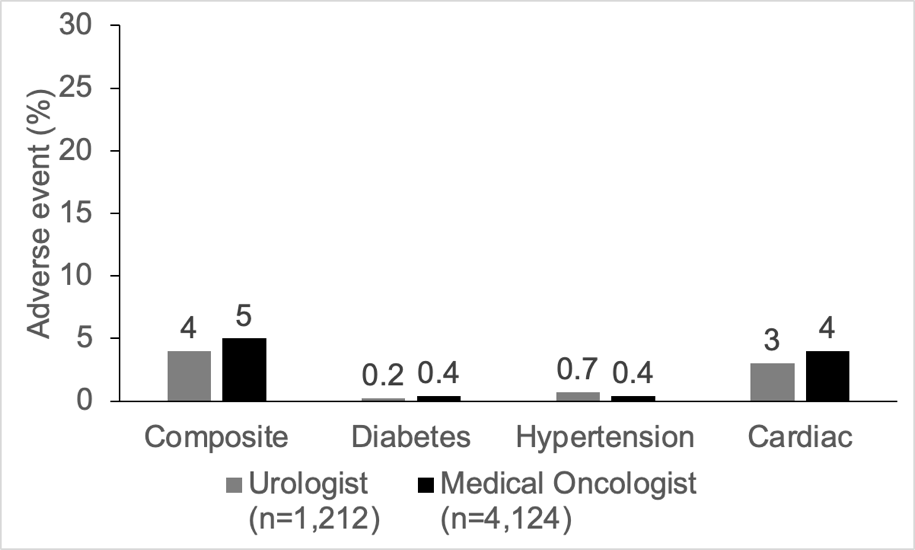


A


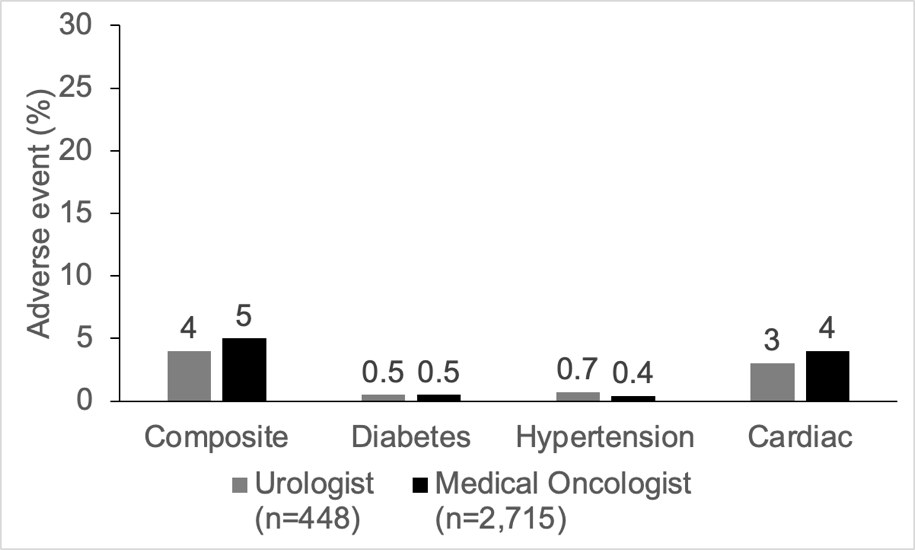


B


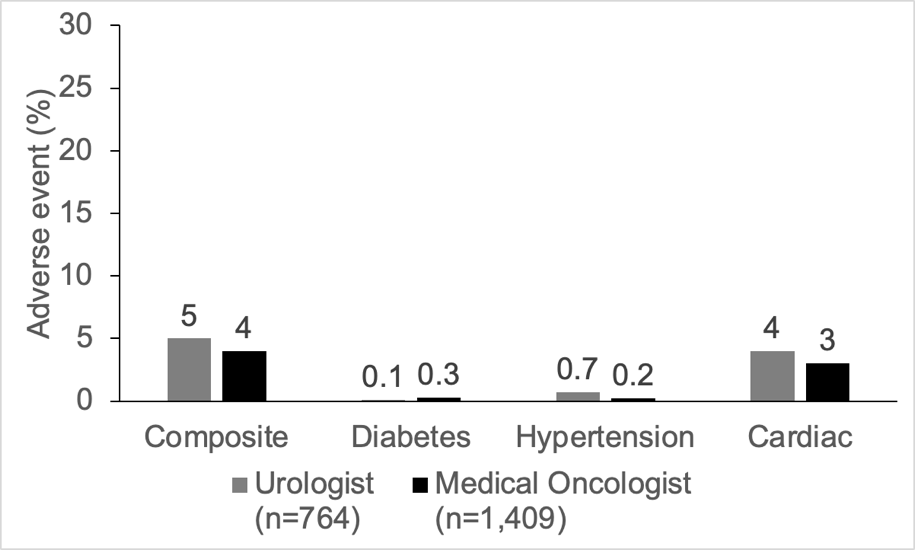


C
