# Supplementary material for: Outcomes of Novel Hormonal Therapies in Men With Advanced Prostate Cancer by Treating Specialist
Source: Cancer Med. 2025 Sep 9;14(17):e71219. doi: 10.1002/cam4.71219 (PMC12417965; doi:10.1002/cam4.71219)
Supplement: Supplementary file 2 — Figure S2: Unadjusted treatment adherence by specialist (*p < 0.05). (A) Entire cohort. (B) By drug type. [file CAM4-14-e71219-s006.docx]

**Supplemental Figure 2**. Unadjusted treatment adherence by specialist (*p<0.05). A) Entire cohort. B) By drug type


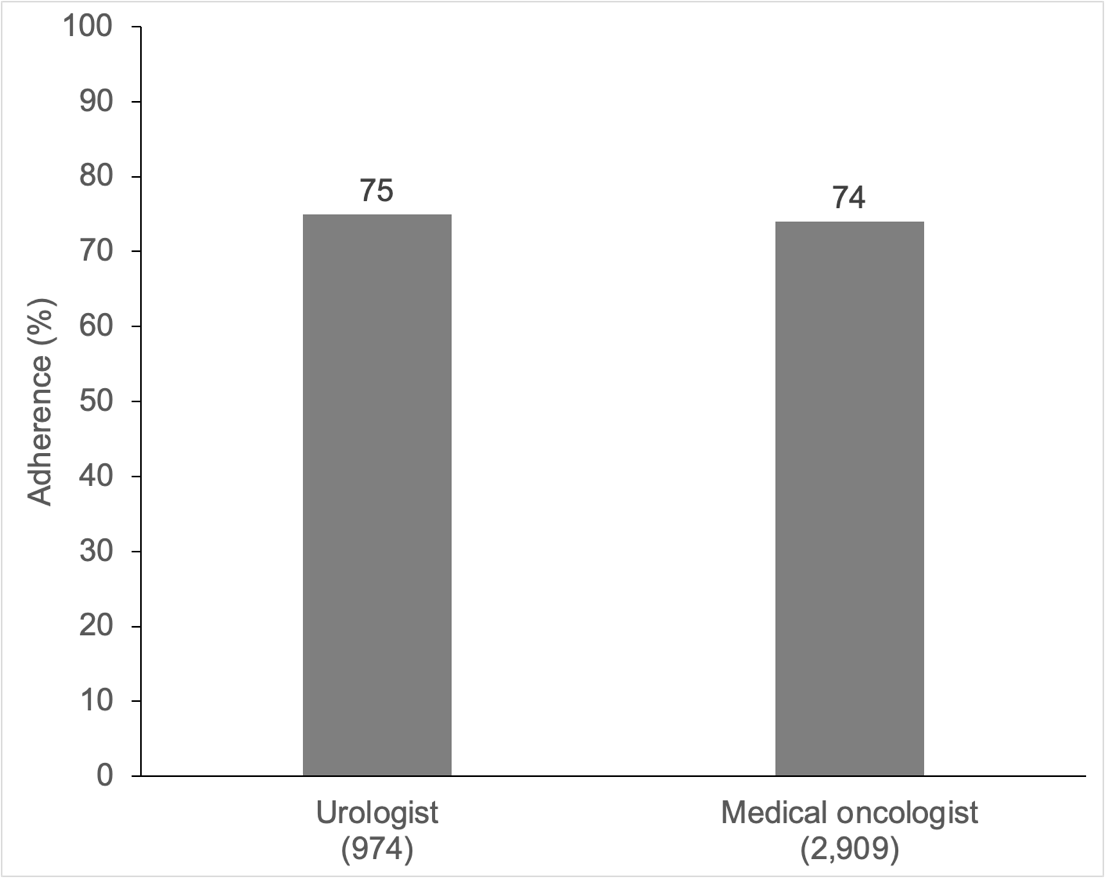


A


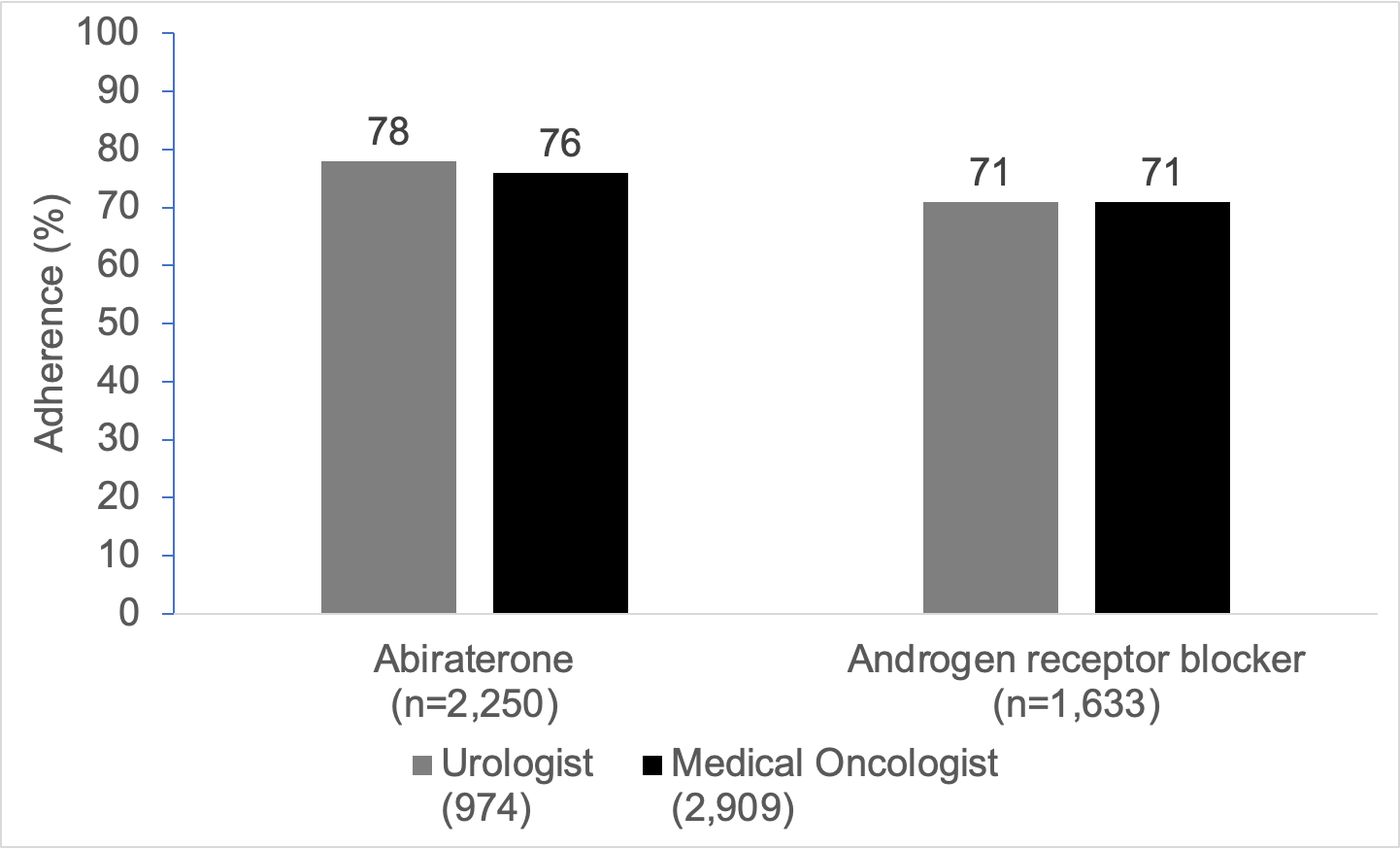


B
