# Supplementary material for: Outcomes of Novel Hormonal Therapies in Men With Advanced Prostate Cancer by Treating Specialist
Source: Cancer Med. 2025 Sep 9;14(17):e71219. doi: 10.1002/cam4.71219 (PMC12417965; doi:10.1002/cam4.71219)
Supplement: Supplementary file 3 — Figure S3: Unadjusted patient out‐of‐pocket costs by specialist in men without low‐income subsidies (*p < 0.05). (A) Entire cohort. (B) By drug type. [file CAM4-14-e71219-s005.docx]

**Supplemental Figure 3**. Unadjusted patient out-of-pocket costs by specialist in men without low income subsidies (*p<0.05). A) Entire cohort. B) By drug type


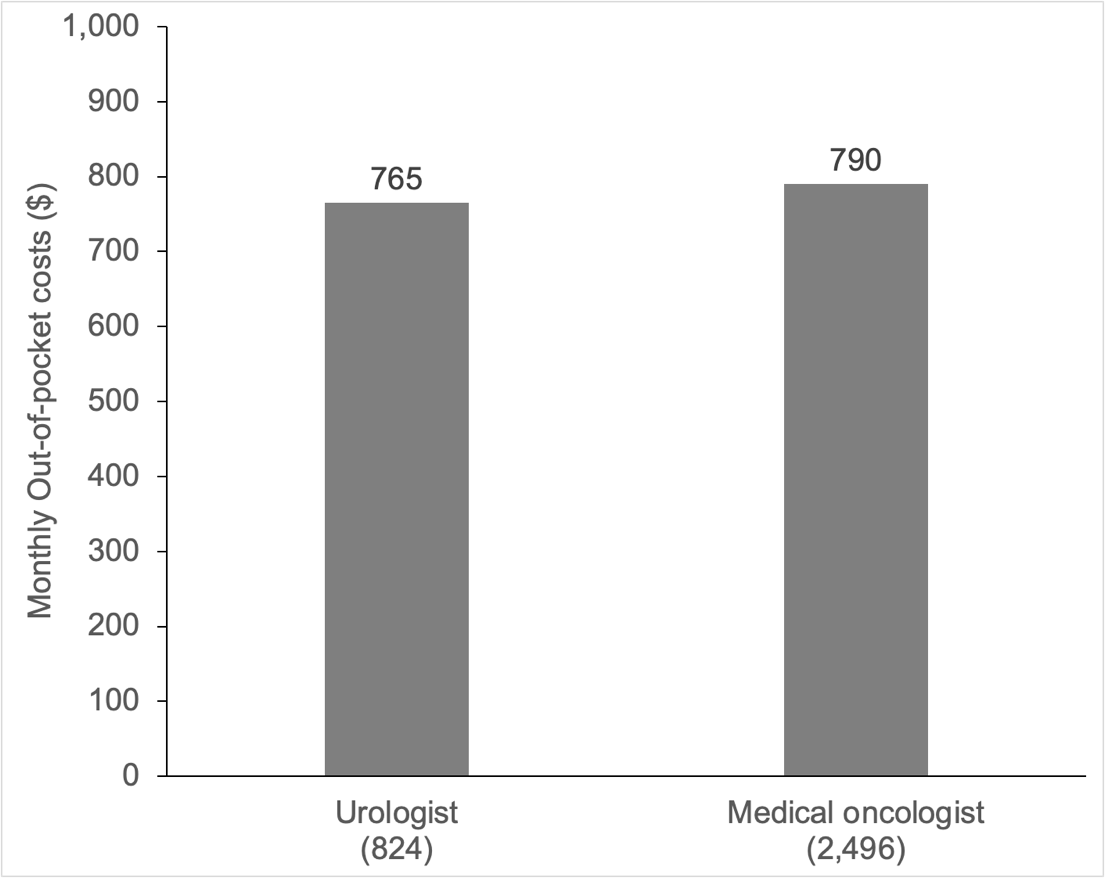


A


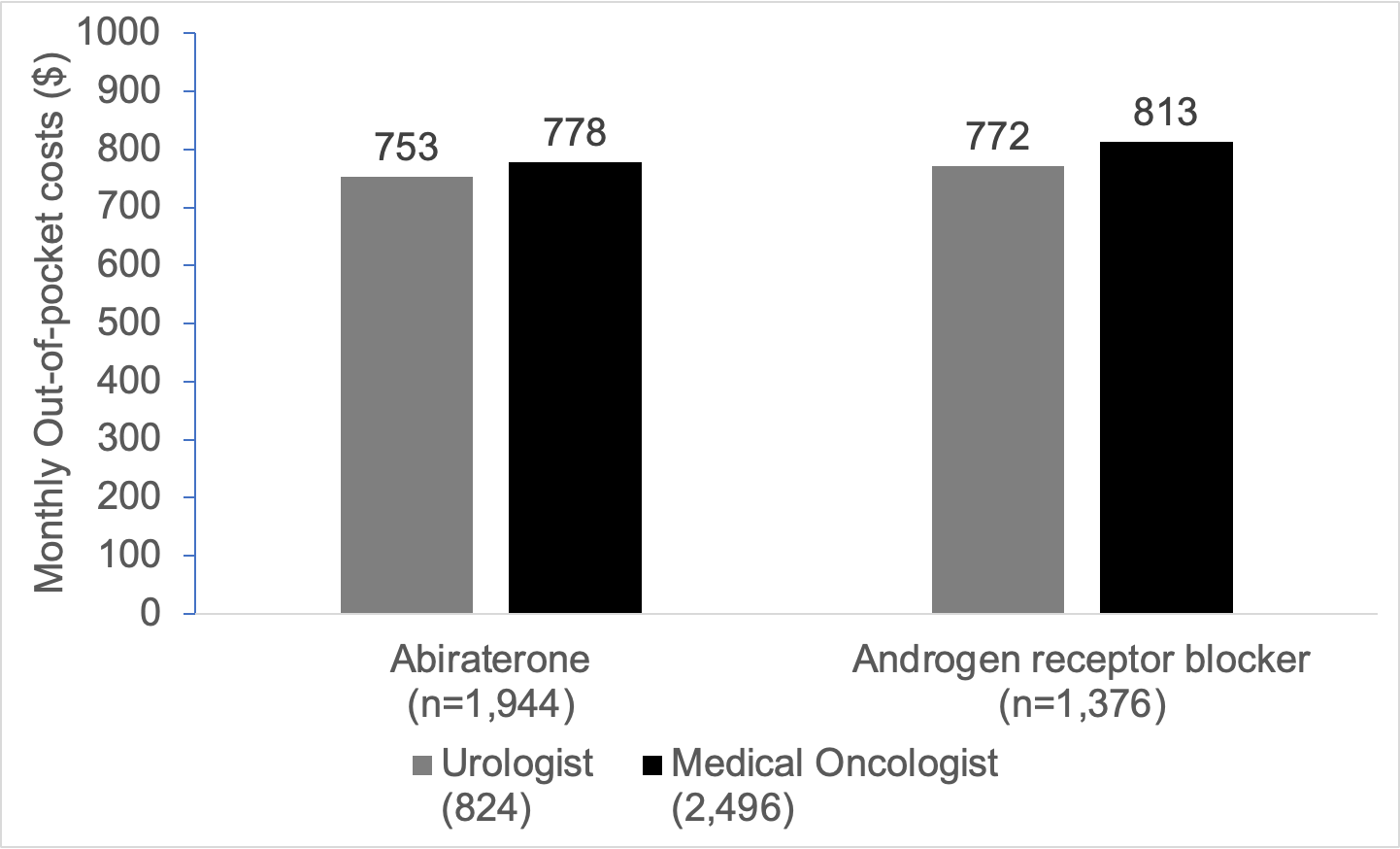


B
