# Supplementary figures and images for: Outcomes of Novel Hormonal Therapies in Men With Advanced Prostate Cancer by Treating Specialist
Source: Cancer Med. 2025 Sep 9;14(17):e71219. doi: 10.1002/cam4.71219 (PMC12417965; doi:10.1002/cam4.71219)

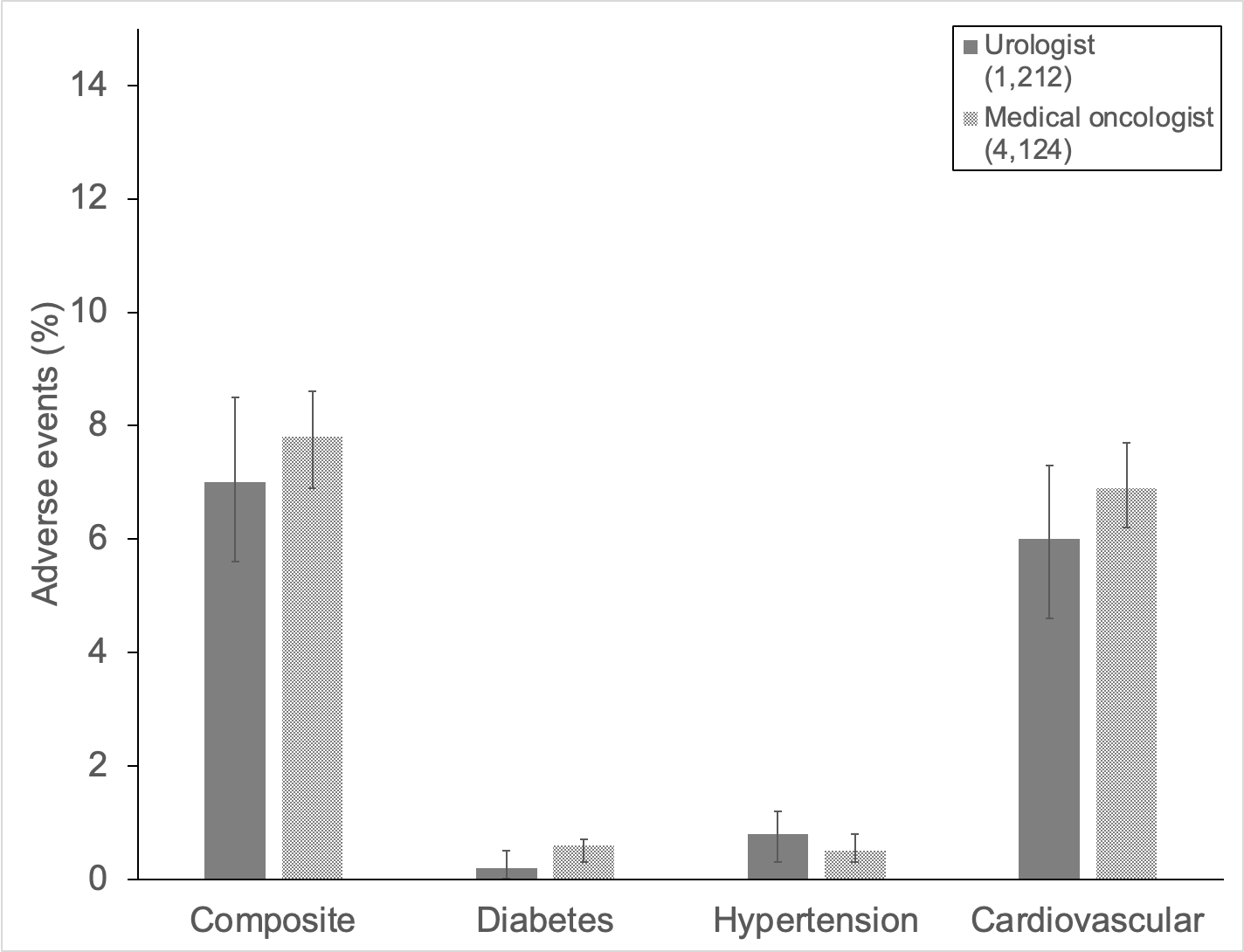

Supplement: Supplementary file 4 — Figure S4: Adverse events by specialty in the 12 months after treatment initiation. There was no difference in the composite adverse event (7% vs. 8%, p = 0.43) or any individual adverse events between specialties. [file CAM4-14-e71219-s002.docx]

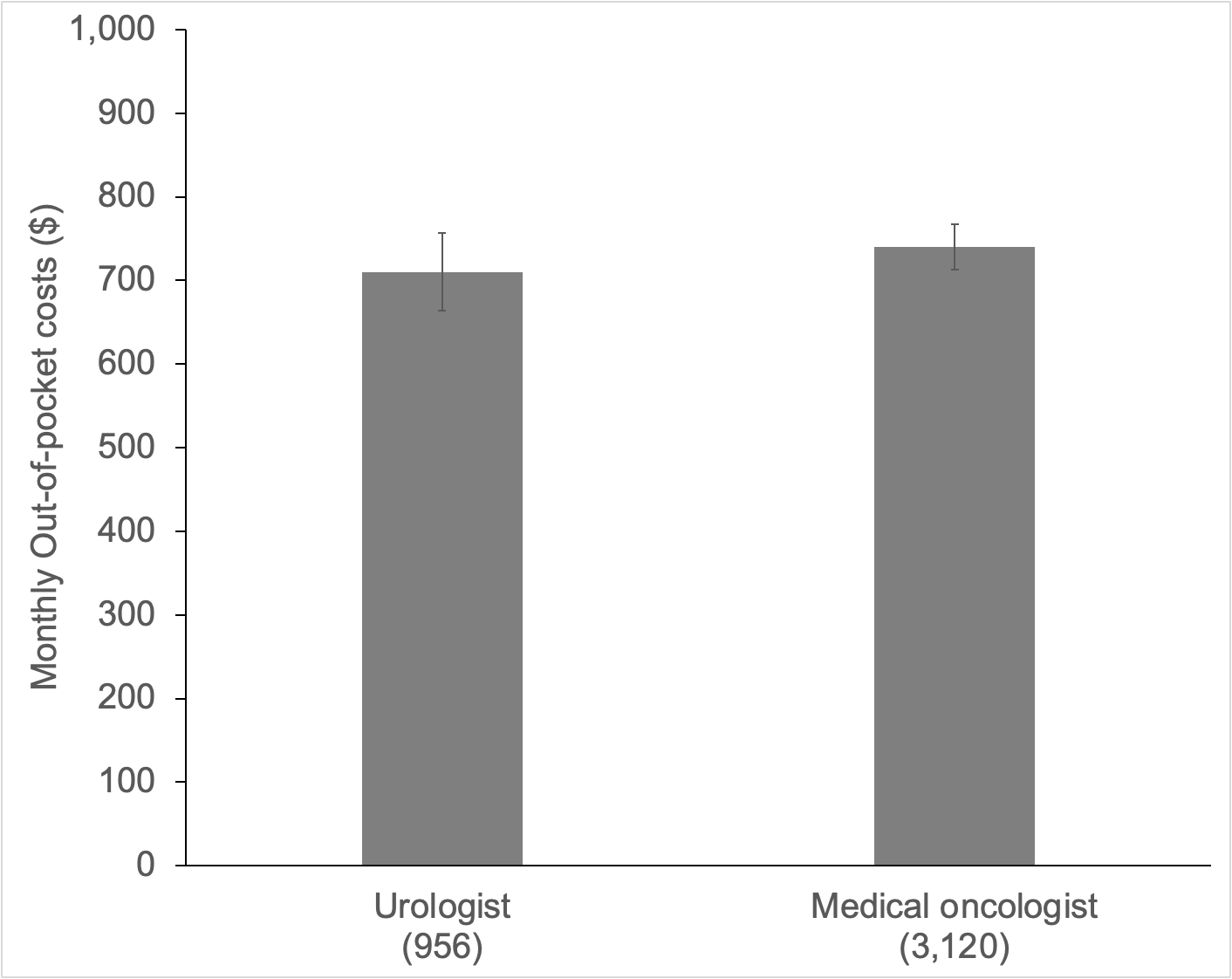

Supplement: Supplementary file 5 — Figure S5: Monthly out‐of‐pocket costs in the 12 months after treatment initiation. There was no difference in out‐of‐pocket costs between specialties ($710 vs. $740, p = 0.30). [file CAM4-14-e71219-s003.docx]

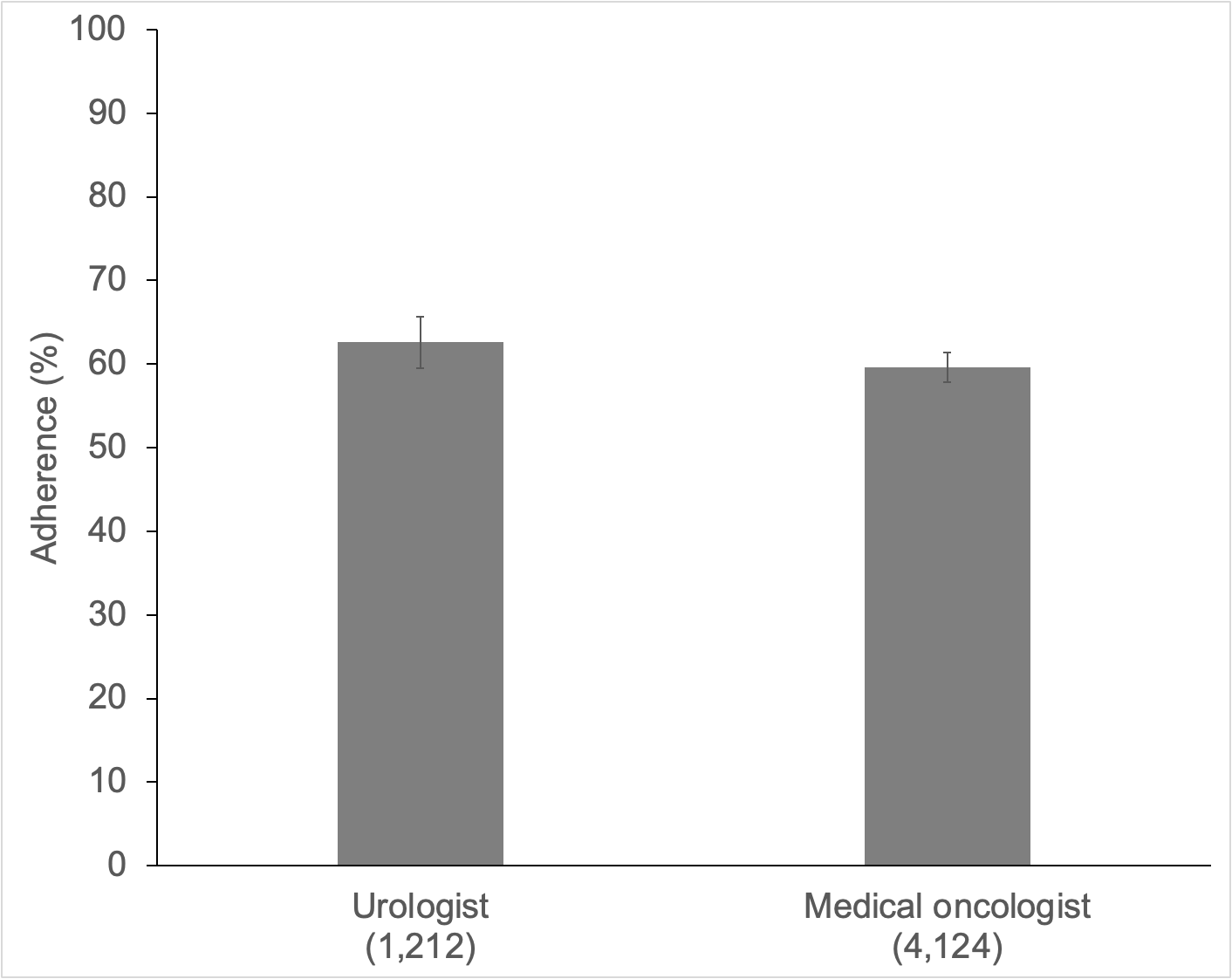

Supplement: Supplementary file 6 — Figure S6: Adherence to treatment in the 12 months after treatment initiation. There was no difference in adherence between specialties (63% vs. 60%, p = 0.20). [file CAM4-14-e71219-s001.docx]
